# HEX: a safe research framework for hybrid EMT X-ray navigation

**DOI:** 10.1007/s11548-023-02917-y

**Published:** 2023-05-12

**Authors:** Henry J. Krumb, Bernhard Dorweiler, Anirban Mukhopadhyay

**Affiliations:** 1 Computer Science Department, TU-Darmstadt, Fraunhoferstr. 5, 64283 Darmstadt, Germany; 2 Vascular Surgery, UK Köln, Kerpener Str. 62, 50937 Cologne, Germany

**Keywords:** Electromagnetic tracking, Radiation reduction, Hybrid navigation

## Abstract

**Purpose:**

Navigating with continuous X-ray provides visual guidance, but exposes both surgeon and patient to ionizing radiation, which is associated with serious health risks. Interleaving fluoro snapshots with electromagnetic tracking (EMT) potentially minimizes radiation.

**Methods:**

We propose hybrid EMT + X-ray (HEX), a research framework for navigation with an emphasis on safe experimentation. HEX is based on several hardware and software components that are orchestrated to allow for safe and efficient data acquisition.

**Results:**

In our study, hybrid navigation reduces radiation by $71\%$ with cubic, and by $40\%$ with linear error compensation while achieving submillimeter accuracy. Training points for compensation can be reduced by half while keeping a similar accuracy–radiation trade-off.

**Conclusion:**

The HEX framework allows to safely and efficiently evaluate the hybrid navigation approach in simulated procedures. Complementing intraoperative X-ray with EMT significantly reduces radiation in the OR, increasing the safety of patients and surgeons.

## Introduction

Continuous fluoroscopic imaging is the gold standard for navigation in endovascular surgery [1]. But although continuous fluoroscopic X-ray supports the mental model of the surgeon by providing visual guidance, associated radiation exposure poses a serious health risk [2]. Moving from continuous fluoroscopy to operator-controlled imaging (OCI) can significantly decrease the dose area product (DAP) and fluoroscopy time (FT) [2, 3]. However, when no X-ray image is available, the surgeon would need to navigate blindly.

We have previously shown that complementing X-ray with electromagnetic tracking (EMT) has the potential to provide a fall-back navigation modality when no X-ray is available, while reducing radiation [4]. X-ray snapshots are taken at discrete time intervals, while EMT is used for continuous navigation. Such *hybrid* setting adheres to the *as low as reasonably achievable* (ALARA) principle [2], as only a minimal number of X-ray snapshots are taken and EMT is used for continuous navigation in between. However, combining X-ray and EMT requires accurate registration strategies and a framework to automate the image acquisition process in real time.

**Fig. 1:**
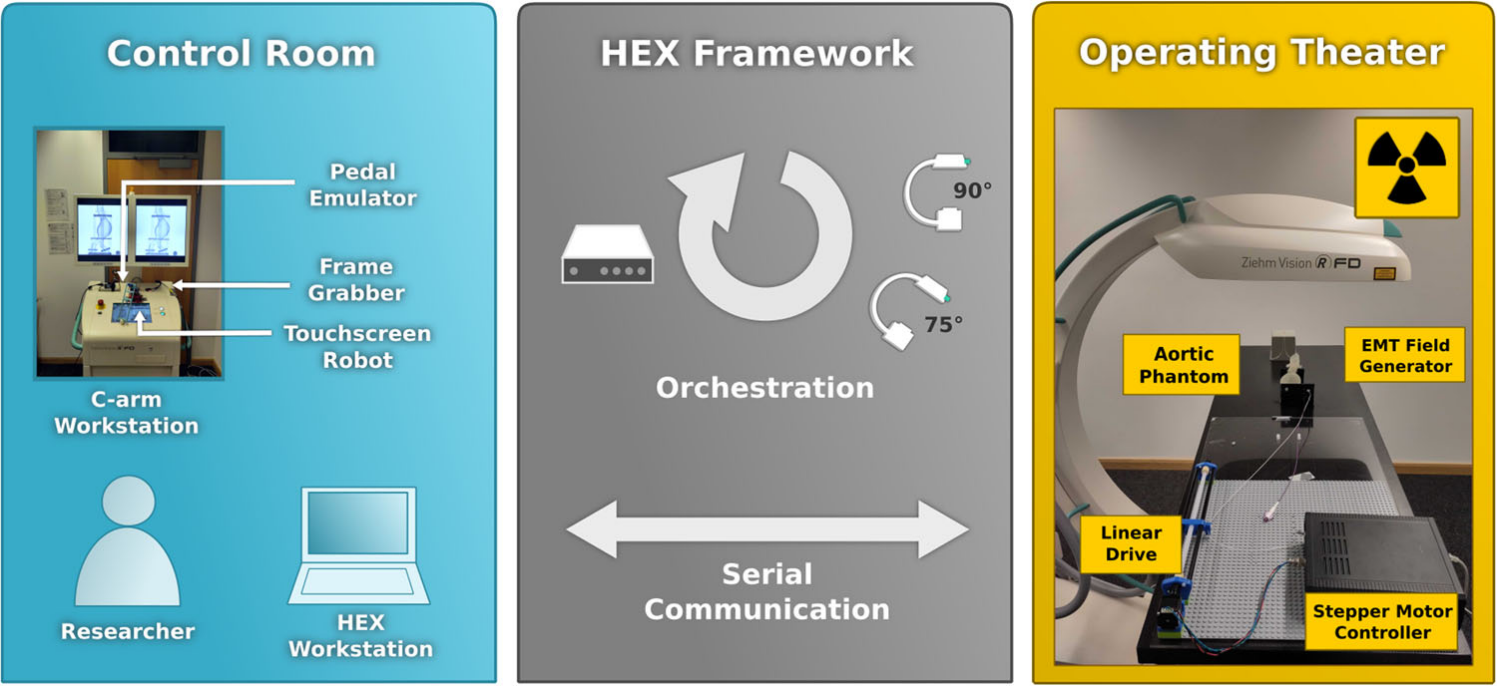
The HEX framework orchestrates the acquisition process used for hybrid navigation in a research setting, allowing for spatial separation of high radiation and safe environments

To our knowledge, a complete *hybrid navigation* approach with EMT and X-ray to reduce radiation has not yet been demonstrated. The idea itself is already discussed in literature [4, 5], but so far only individual components of the hybrid pipeline are evaluated whereas the actual radiation saving of the full pipeline has not yet been investigated.

In this paper, we implement a complete hybrid setup by presenting the hybrid EMT + X-ray (HEX) framework, a safe research environment to investigate hybrid navigation in minimal invasive surgery. One major obstacle that we overcome in this paper is the radiation safety with regards to the researcher who needs to acquire large datasets for retrospective evaluations. In the longer run, the foundational research enabled by HEX will be translated to the operating theater.

Our contributions are threefold:

1. We are the first to analyze the trade-off between radiation exposure and tracking accuracy in hybrid navigation with EMT and X-ray. Further, we analyze the impact of individual parts of the pipeline on the overall accuracy and radiation, evaluating exemplary compensation mechanisms.
2. We present the HEX research framework, which is the first of its kind to assess the feasibility of a hybrid navigation setting in phantom studies. HEX will be published under a free software license after acceptance.
3. The HEX framework features several means of automation that allow for safe but efficient experimentation. Large amounts of data can be recorded without researchers having to expose themselves to radiation.

## Related work

Complementing fluoroscopic navigation with EMT is an idea that is discussed in literature [4, 5], and several optimizations have been proposed to improve the accuracy of EMT in the hybrid setting. For instance, distortion compensation on the hardware level is investigated by the group around the Anser EMT project, which is intended to be used in the vicinity of the C-arm [5, 6]. One optimization involves a field generator that is designed to be radiolucent in order to simplify hybrid navigation under X-ray [7]. These optimizations are intended to be leveraged in hybrid navigation with EMT and X-ray imaging.

While integration EMT with ultrasound is common [8], only few related works cover the integration of EMT and X-ray imaging in a hybrid setup, as the registration is more difficult [9]  or requires dedicated hardware [10].

Our previous work analyzes tracking accuracy in the hybrid setting in in-silico and in-vitro studies [4, 11, 12] and discusses techniques for algorithmic EMT distortion compensation. An overview of EMT systems, as well as previous passive and active techniques to mitigate EMT distortion, is found in the comprehensive review by Franz et al. [13].

So far, research has focused on optimizing individual parts of the hybrid setting, but a complete framework for hybrid navigation with EMT and X-ray is not yet available to the research community. Analyzing the impact of such modifications *on radiation reduction* in the hybrid setting is therefore not possible. With *HEX*, we introduce the first complete open-source framework that enables an ablation of individual parts of the pipeline on the overall navigation accuracy.

## Methods

**Fig. 2:**
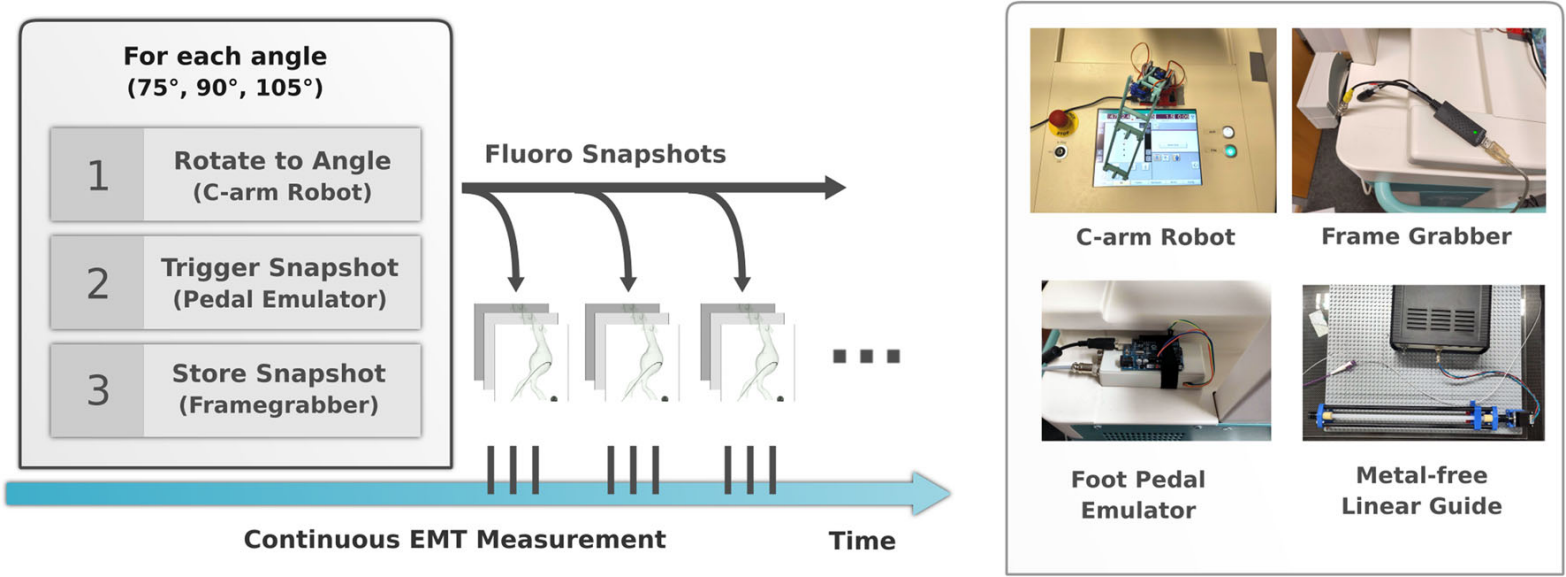
Left: Orchestration of continuous EMT and fluoro snapshots. Three fluoro snapshots are taken automatically, in order to determine the 3D pose of the sensor. In between snapshot triplets, EMT is used for continuous navigation. Right: Hardware components we use to automate C-arm and EMT data acquisition

Ensuring safety for researchers in a laboratory environment requires the interplay of different automated devices in spatially separated rooms (see Fig. 1). With such an automated setup, it is possible to generate large amounts of data for evaluation in retrospective. HEX comprises several hardware components (see Fig. 2) and a software/firmware framework to orchestrate those, which together provide a safe and efficient data acquisition routine. We will first outline our imaging pipeline for EMT to X-ray registration and then elaborate on our automation approach. Finally, we will detail the evaluation protocol and materials and data used in our study.

3D EMT points are registered to X-rays by taking images from three gantry orientations, namely $75^\circ$, $90^\circ$ and $105^\circ$. Using the known angular difference between both images, the 3D position of the sensor is deduced in image coordinates (we assume the coordinate frame to be in the center of the $90^\circ$ image) by modeling the C-arm as a pinhole camera. This process requires a detection framework to determine the EMT position on X-rays, as well as a calibration routine for the C-arm. Finally, the EMT sensor data are aligned into the coordinate frame of the image and are recalibrated when a set of new images is taken.

### EMT sensor detection on X-ray

**Fig. 3:**
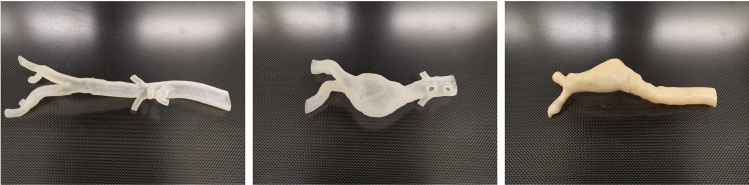
3D-printed phantoms based on real patient anatomy. Left: healthy aorta (flexible, translucent), center: abdominal aneurysm (flexible, translucent), right: abdominal aneurysm (rigid, opaque)

Detecting the EMT sensor on X-ray is challenging due to the noisy acquisition process. We therefore use the YoloV5 detection network [14], as Yolo is successfully employed in both real-world and medical applications (e.g., ultrasound [15]). The weights we use are pre-trained on MSCOCO, re-trained on 154 and validated on 60 hand-annotated images with EMT sensors for 300 epochs, yielding 0.955 mAP50 and 0.512 mAP50-95 [16]. During detection, we consider the center of the detected bounding box in each image as the sensor position (Fig. 3).

### C-arm calibration

**Fig. 4:**
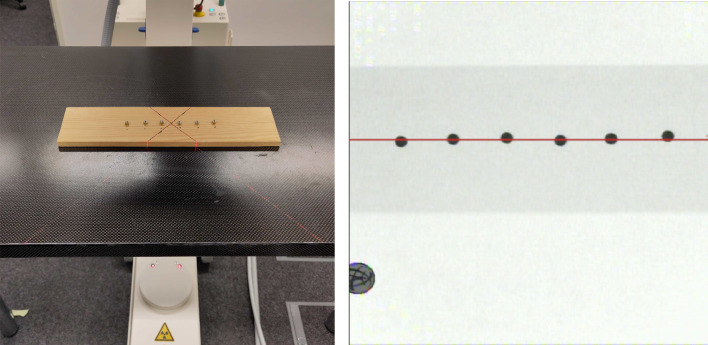
Custom C-arm calibration board under camera (left) and X-ray (right), at a gantry height of $10\,{\text {cm}}$ and an angle of $75^\circ$. Red line indicates the average center of the metallic orbs, which is used to determine the pivot point of the gantry

For registering 3D EMT points to the X-ray, it is necessary to find the center of rotation in an image depending on the gantry angle and height at which it was captured. In clinical practice, this calibration step would be executed *once* for all subsequent interventions. We use a polynomial regression approach in order to infer the center of rotation, which we obtain by capturing images of a custom calibration board with metallic orbs, which is shown in Fig. 4. The orbs are positioned on a straight line that is aligned to the C-arm’s laser beams. Then, each orb is detected using the OpenCV built-in blob detector. At minimum three orb centers per image are then averaged to find the x-coordinate at which the C-arm gantry pivots with respect to the image taken at $90^\circ$. Finally, we fit a family of polynomials to all pivot centers depending on the gantry height, to obtain a model for the pivot center depending on gantry height and angle.

### EMT to image registration

With the detected 2D sensor positions in three images, we estimate the sensor’s 3D position by calculating the intersection point between orthogonal rays through the images. We obtain two sets of 3D points: one from the sensor itself and one from the detected positions on X-ray. These two point sets are aligned by rigid registration.

### Automated X-ray acquisition protocol

Two prerequisites are necessary to automatically take X-ray snapshots from different angles: C-arm gantry rotation and X-ray acquisition. HEX automates both tasks, ensuring a safe procedure for the researcher and time-efficient collection of data for later evaluation.

**Fig. 5:**
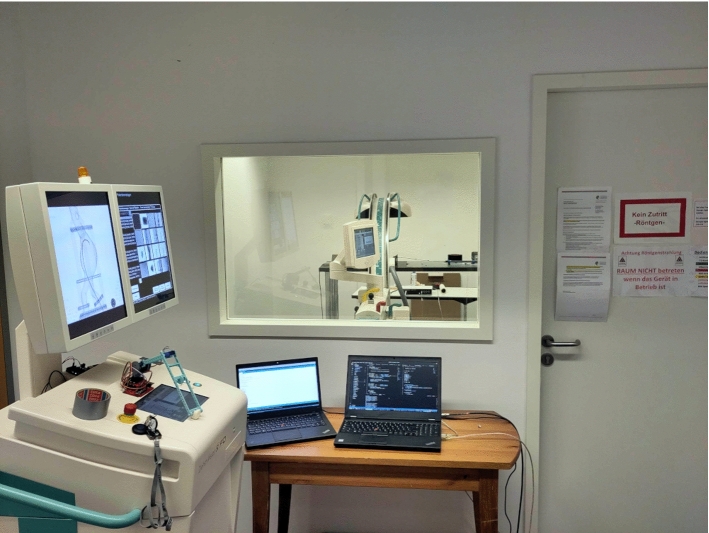
Workstation (left) and C-arm with phantom setup (center) are spatially separated by a lead-supported wall to block radiation from the researchers inside the control room

In the OR, the rotation is carried out either by manually rotating the gantry or by pressing a virtual button on the resistive touchscreen of the C-arm workstation. Since there is no accessible digital interface available, the easiest solution to automate the rotation is to press the touchscreen with the help of a robot. We have developed a lightweight 3D-printed 3-axis robot to accomplish this task (Fig. 5). To control the gantry, we determined the setup time and angular velocity of the C-arm gantry to be $t_{\text {setup}} = 1.0\,{\text {s}}$ and $\omega _{\text {gantry}} = 4.0 \frac{\circ }{s}$.

**Fig. 6:**
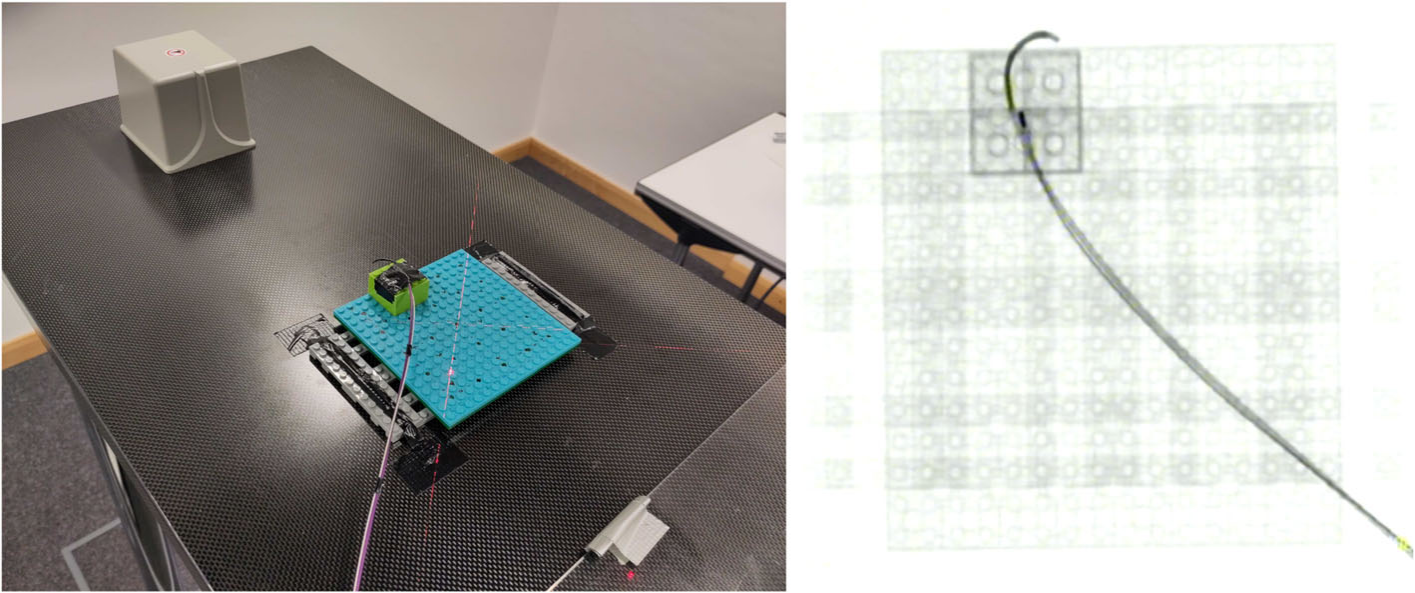
Left: Lego calibration board and sensor fixture for EMT compensation. $25\times 25$ training points are marked in a grid, whereas validation points are taken arbitrarily. Right: Same Lego board under X-ray

Once the C-arm gantry is set to the desired orientation, the image acquisition needs to be triggered. The designated way to capture images with the Ziehm Vision RFD is to manually trigger the foot pedal. As the foot pedal merely closes a switch, we replaced it with an Arduino-controlled relay which is triggered for a certain amount of time. We found that $100\,{\text {ms}}$ is the minimum exposure time to obtain a good quality X-ray image. Once triggered, X-rays are taken from the C-arm’s analog video output using a USB framegrabber, averaging over 100 frames to eliminate noise in the analog signal. HEX also provides access to higher resolution DICOM images if the C-arm is connected to a Picture Archiving and Communication System (PACS).

In between X-ray snapshots, the EMT sensor, which is attached to a directed catheter, is moved under the use of a 3D-printed linear guide. The sensor is retracted in steps of $2\,{\text {mm}}$ while taking positional measurements with the EMT system (Fig. 6).

**Table 1:** List of datasets we use for individual parts of our data-driven pipeline

Purpose	#Images	#EMT Points	Anatomy
Pivot point	60	N/A	Calibration Board
Pixel to mm conversion	10	N/A	Caliper
Sensor detection train/val/test	154/60/14	N/A	Aneurysm, Lego Board
EMT compensation train/test	100/32	46,229/12,857	Lego Board
Final evaluation	180	52,278	Healthy Phantom
Total	600	111,364	

### EMT distortion compensation

An EMT distortion compensation scheme is implemented, for which we collect a total number of 132 points on a Lego board (100 training, 32 validation), with the gantry being rotated to a fixed angle of $90^\circ$. As ground truth, we use the corresponding detected sensor positions on the X-rays. Both linear and cubic compensation schemes are implemented and evaluated.

It must be noted that this kind of compensation is an *offline* compensation scheme, which would require preoperative data collection in the OR, which typically is barely feasible. Online compensation, which has been proposed earlier [12], eliminates one more calibration step in clinical practice. However, in this work, we only apply exemplary *offline* compensation mechanisms for the sake of simplicity and brevity  [12].

### Materials

We use an Ascension/NDI trakSTAR 3D Guidance EMT navigation system (180-type sensor, mid-range field generator). HEX communicates with the tracker via OpenIGTLink, such that other trackers can be plugged in with ease. X-ray snapshots are taken with a Ziehm Vision RFD C-arm. All software modules are written in Python and C++ under the use of Pytorch and OpenCV libraries.

## Experimental setup and results

We detail the experiments conducted with our hybrid pipeline and the resulting intermediate findings. Finally, we evaluate our phantom study, which investigates the effect of EMT compensation on overall radiation saving.

### Data acquisition protocol

Accuracy is evaluated on a dedicated test measurement with the sensor and catheter inside the healthy phantom (see Fig. 3), with which $3\times 60$ snapshots are collected. In this measurement run, the sensor is moved in 60 steps with a step size of $2\,{\text {mm}}$. To evaluate accuracy subject to radiation exposure, we gradually reduce the number of snapshots inside the dataset in retrospective. Each snapshot-triplet ($75^\circ$, $90^\circ$, $105^\circ$) is then replaced by the corresponding EMT position from the timestamp of the $90^\circ$ image. Error is calculated as the difference between the registered EMT point and the detected sensor position on the corresponding X-ray. We estimate the conversion factor from X-ray pixels to mm by taking X-rays of a caliper at defined lengths and measuring the corresponding sizes in pixels.

**Table 2:** Comparison of sensor detection models

Model	Size	mAP50	mAP50-95
YoloV5-s	7 M	0.913	0.502
YoloV5-l	46 M	0.948	0.516
YoloV5-x	86 M	**0**.**959**	**0**.**530**

‘Size” is the number of trainable parameters of the final model (in million)

**Table 3:** Test set error (in mm) of compensation models

	None	Linear	Cubic
$E_X$	2.04	0.27	**0**.**26**
$E_Y$	1.68	0.31	**0**.**18**
$E_Z$	23.10	0.25	**0**.**23**
RMSE	13.42	0.28	**0**.**23**

**Fig. 7:**
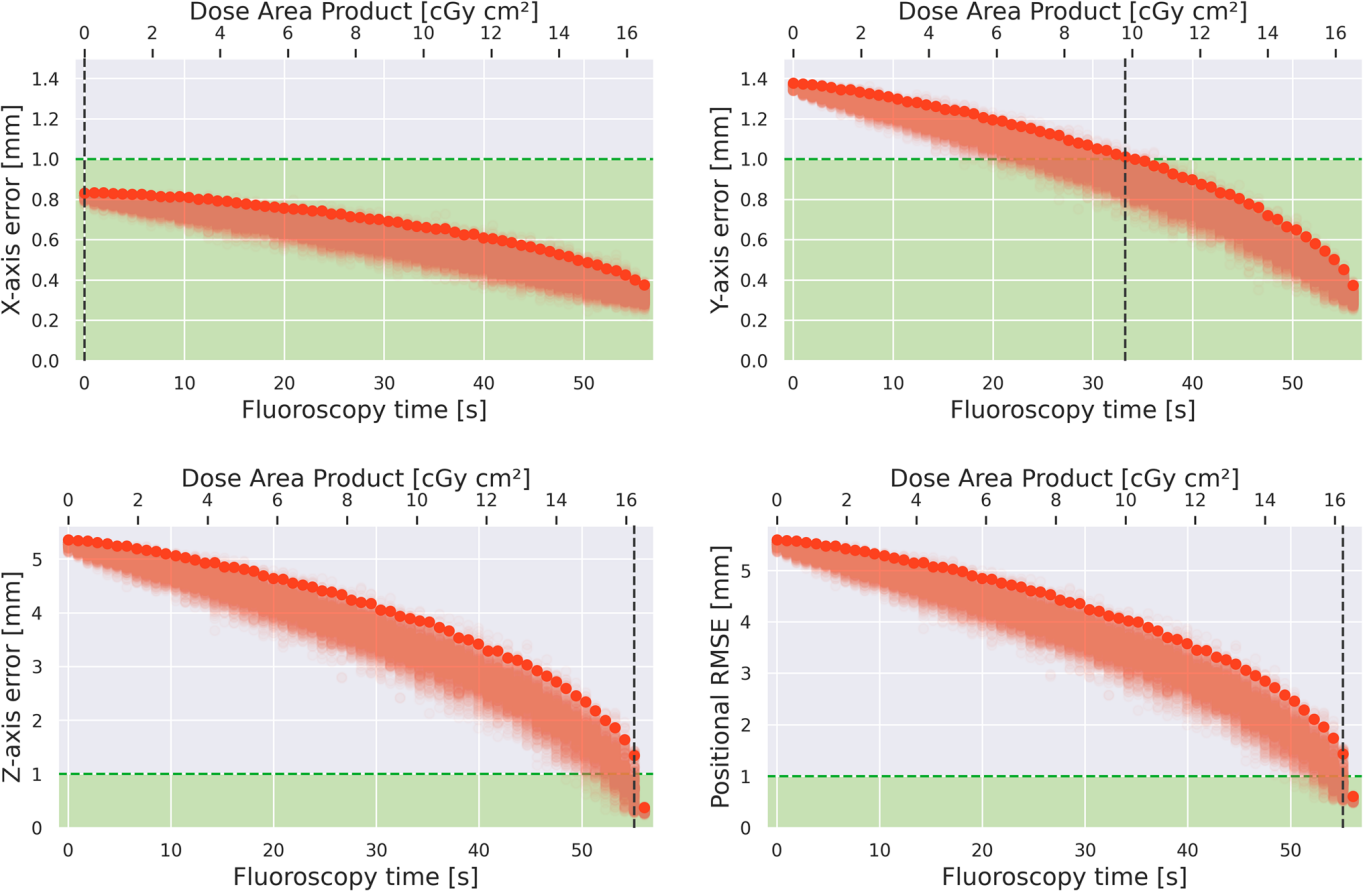
Tracking error (X, Y, Z, total RMSE) over FT and DAP *without EMT distortion compensation*. Dark points mark the CI-95 of samples, which we interpret as the Pareto Front between radiation exposure and accuracy. Green region marks the $1\,{\text {mm}}$ requirement for high-precision surgery, whereas the vertical line marks the FT at which $1\,{\text {mm}}$ is reached

**Fig. 8:**
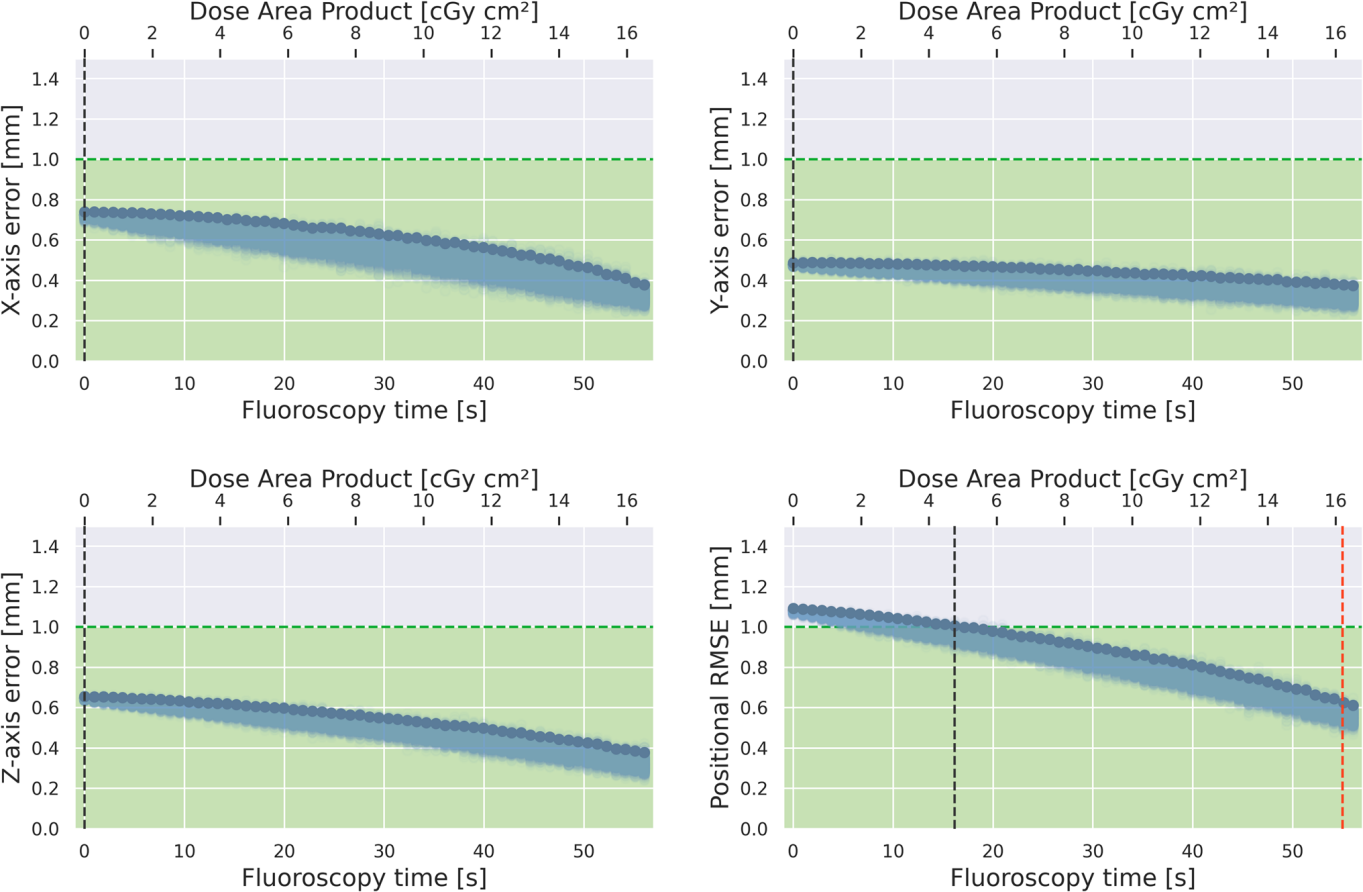
Tracking error over FT and DAP *with cubic EMT distortion compensation*. For comparison, the red line marks the radiation–accuracy sweet spot without compensation

**Fig. 9:**
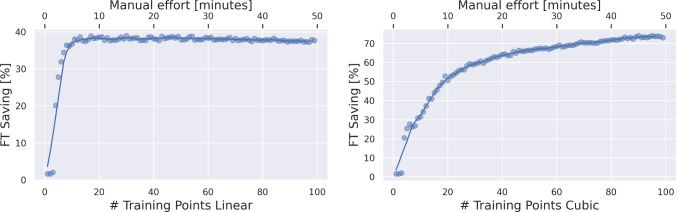
Radiation (FT) saving in percent over number of training points for linear (left) and cubic (right) compensation, determined in Monte Carlo experiment with 1000 iterations

### Datasets

Parts of the HEX pipeline, as well as the final evaluation (“Experimental setup and results” Section), rely on pre-recorded data. A summary of these datasets and their respective purpose can be found in Table 1. In total, we have collected 600 X-rays and over 100k EMT points for the experiments conducted in this study. We plan to release these datasets together with our code upon acceptance.

### Analysis of intermediate results

Tables 2 and 3 show intermediate results for trainable sensor detection and EMT compensation, respectively. The evaluation of the accuracy vs. radiation trade-off we described earlier uses the best-performing models for each task.

### Evaluation protocol

Radiation exposure is evaluated in phantom studies with three 3D-printed anatomical vascular phantoms, which are shown in Fig. 3. We state the total FT and DAP [2] for the simulated guidewire retractions to indicate the achieved radiation reduction. It must be noted that these values not do not directly quantify the exposure to surgeon or patient—evaluating this would add significantly to the complexity of our setup. The C-arm itself reports an average FT of $0.95 \pm 0.39\,{\text {s}}$ and an average DAP of $0.28 \pm 0.12~cGy\,{\text {cm}} ^2$ per X-ray for the parameters we set (48 kV, 2.6 mA, 57% pulse width, 25 pulses/s). FT and DAP were averaged over 20 X-ray snapshots taken with our setup, and the average values are multiplied with the overall number of X-rays taken in each measurement run.

### Phantom study

The main goal of HEX is to minimize total radiation exposure, while maintaining reasonable accuracy. Figures 7 and 8 visualize this trade-off in a Monte Carlo experiment, in which we leave out $M={0...60}$ random images from the X-ray set, replacing them with the corresponding EMT point. For the remaining X-rays, we model the navigation error as a Gaussian with $\sigma =0.32\,{\text {mm}}$, corresponding to the pixel quantization noise. We repeat this process for $N=200$ iterations and evaluate for uncompensated and compensated (cubic) EMT data. This allows us to visualize total navigation accuracy over the number of X-rays taken during a procedure, the latter being proportional to the total FT or DAP, respectively.

This way, we observe that EMT distortion compensation has a beneficial effect on the overall tracking accuracy of the hybrid pipeline, achieving submillimeter accuracy even for a small number of snapshots. If we assume $1\,{\text {mm}}$ to be the upper bound for high precision accuracy, hybrid navigation with cubic offline distortion compensation reduces FT from $60\,{\text {s}}$ down to $17.1\,{\text {s}}$, limiting overall radiation exposure by $71\%$. Linear distortion compensation reduces FT by $36\,{\text {s}}$ ($40\%$), whereas submillimeter accuracy is not achieved without compensation. Further, we analyze the impact of the number of training points on the radiation saving, as these are collected manually which is tedious (see “EMT distortion compensation” Section). It is possible to reduce the training points by half, while still saving more than $70\%$ of radiation exposure.

## Conclusion

In this paper, we demonstrate how an end-to-end pipeline for hybrid navigation can be realized in a research environment. Combining custom hardware and off-the-shelf components, we complement fluoroscopy by EMT guidance, which effectively reduces the radiation dose. The automated image acquisition protocol allows us to collect large amounts of data safely and efficiently, with a total of 600 X-ray images being used in the scope of this paper. Efficient data collection enables analyses of the impact of optimizations on the full pipeline before deployment of optimizations in the OR. A phantom study shows that even with a simple compensation scheme, we are able to reduce fluoroscopy time by $71\%$. This result indicates that HEX can significantly decrease radiation exposure to patients and surgeons and should be investigated toward bench-to-bedside translation in endovascular surgery.

## Data Availability

The code used in this work will be made available via https://github.com/ai-health-care/Hybrid-EMT-Xray upon acceptance.
